# Bioinformatics in Italy: BITS 2012, the ninth annual meeting of the Italian Society of Bioinformatics

**DOI:** 10.1186/1471-2105-14-S7-S1

**Published:** 2013-04-22

**Authors:** Carmela Gissi, Paolo Romano, Alfredo Ferro, Rosalba Giugno, Alfredo Pulvirenti, Angelo Facchiano, Manuela Helmer-Citterich

**Affiliations:** 1 Dipartimento di Bioscienze, Università degli Studi di Milano, Milano, I-20133, Italy; 2 IRCCS AOU San Martino IST, Genova, I-16132, Italy; 3 Department of Clinical and Molecular Biomedicine, University of Catania, Italy; 4 CNR Istituto di Scienze dell'Alimentazione, Avellino, Italy; 5 Department of Biology, University of Rome "Tor Vergata", Rome, I-00133, Italy

## Abstract

The BITS2012 meeting, held in Catania on May 2-4, 2012, brought together almost 100 Italian researchers working in the field of Bioinformatics, as well as students in the same or related disciplines. About 90 original research works were presented either as oral communication or as posters, representing a landscape of Italian current research in bioinformatics.

This preface provides a brief overview of the meeting and introduces the manuscripts that were accepted for publication in this supplement, after a strict and careful peer-review by an International board of referees.

## Preface

### The Italian Society of Bioinformatics

The Italian Society of Bioinformatics (BITS) [1] was born in 2003 by initiative of a small group of Italian scientists, involved in various disciplines ranging from physics to informatics and molecular biology. Since then, the number of researchers joining BITS was continuously increased year after year. The Society has now about 250 members, including both young and senior scientists, working in Italy and abroad.

Main aim of the Society, which is a Regional group of the International Society for Computational Biology (ISCB), is the fostering of Bioinformatics in Italy. Its activities include the organization of an Annual Scientific meeting, the maintenance of a web site [1] and of a mailing list for the distribution of news of interest for the community of researchers interested in bioinformatics (more than 700 addresses), the coordination of educational initiatives in Italy, from bachelor to PhD degrees, the coordination of research activities among members, and the improvement of the participation of Italian researchers, both senior and junior, to international events and projects of relevance.

### The Ninth BITS Meeting: BITS 2012

The Ninth Annual BITS Meeting, BITS 2012, was held in Catania kindly hosted by the Department of Mathematics and Computer Science of the University of Catania (Cittadella Universitaria) on May 2-4, 2012 [2]. The University of Catania was founded in 1434 and among its famous scholars had Napoleone Ferrara, molecular biologist, winner of the 2010 Lasker-DeBakey Clinical Medical Research Award.

The meeting was organized by Alfredo Ferro, Rosalba Giugno and Alfredo Pulvirenti. Over 90 scientists actively working in bioinformatics and related fields or strongly interested in its development met and discussed their work, state of the art and future perspectives. A total of 88 abstracts were accepted: 33 of them were selected for oral presentation by the Scientific Committee after a well established peer-review procedure based on three reviews and scores per paper. The remaining 55 were presented in the poster sessions.

Three keynote talks were given by distinguished scientists: Prof. Charles E. Lawrence (Brown University), Prof. Eugene Myers (HHMI Janelia Farm Research Campus and Max Planck Institute for Cellular Molecular Biology and Genetics) and Prof. Ileana Zucchi (CNR, ITB Milano).

Prof. C. E. Lawrence presented interesting applications on Genomics and RNA structure analysis based on statistical inference in high dimensional spaces. Prof. E. Myers gave the Preparata Lecture and talked about bioimage informatics. He showed novel approaches based on a combination of knowledge coming from biophysics and computer science, which allows observing molecular mechanisms within the cell, to look at the developmental of trajectory of growing organs, and to map the cellular anatomy of organisms and organs such as the brain, the heart, or the stem of a plant.

Prof. I. Zucchi gave the Dulbecco Lecture in honour of Professor Renato Dulbecco, who recently passed away. She was a former student of Prof. Dulbecco, Nobel Prize in 1975 for Physiology or Medicine, and talked about his scientific interests and career, his brilliant and innovative ideas and some personal memories.

The conference was organized into thematic sessions that reflected several topics: Genomics, Molecular Evolution, Comparative Genomics, Protein Structure and Function, Proteomics, Transcriptomics, Metagenomics, Systems Biology, Biological Databases, Biobanks, Algorithms for Bioinformatics, Pharmacogenomics, Next Generation Sequencing. One tutorial was also given on the last day of the meeting. Giovanni Micale, from the Department of Computer Science of the University of Pisa, gave a tutorial on "Biological network alignment".

### The selection of papers for this supplement

Shortly after the conference, 18 papers were submitted for publication in this *BMC Bioinformatics* supplement. An Editorial Board was formed, including all members of BITS 2012 Programme Committee.

Associated Editors are listed here:

• Francesca Ciccarelli, European Oncology Institute, Milan, Italy

• Domenica D'Elia, CNR Institute for Biomedical Technologies, Bari, Italy

• Diego Di Bernardo, Telethon Institute of Genetics and Medicine, Naples, Italy

• Angelo Facchiano, CNR Institute of Food Sciences, Avellino, Italy

• Alfredo Ferro, University of Catania, Italy

• Rosalba Giugno, University of Catania, Italy

• Manuela Helmer-Citterich, University of Rome "Tor Vergata", Rome, Italy

• Sabino Liuni, CNR Institute for Biomedical Technologies, Bari, Italy

• Roberto Marangoni, University of Pisa, Pisa, Italy

• Stefano Pascarella, University of Rome "La Sapienza", Rome, Italy

• Alfredo Pulvirenti, University of Catania, Italy

• Paolo Romano, IRCCS AOU San Martino IST, Genova, Italy

A stringent reviewing procedure was then adopted. Associate Editors handled the process according to their recognized knowledge in specific meeting topics. Mainly three referees, with a high reputation at international level, were selected for each submission. Overall, 51 referees from 17 different countries were involved in the selection of papers. We opted for a two-step peer review procedure, offering authors the possibility to submit a new version of their paper, revised according to the referees' comments.

At the end of this process, 13 papers were accepted and are now included in this supplement. They cover different aspects of theoretical and applied Bioinformatics. For the sake of readability, they are presented in this supplement grouped by topics.

### New algorithms

The study entitled "A novel biclustering algorithm for the discovery of meaningful biological correlations between micrornas and their target genes", by Pio et al. [3] proposes a novel data mining algorithm aimed at identifying (a) highly interconnected miRNAs-target modules and (b) novel miRNA targets. The method exploits the bicluster algorithm and is based on experimentally-verified as well as on predicted interactions between miRNAs and RNA targets. The Authors show that their method succeeds in extracting cohesiveness-preserving biclusters and show that mRNAs in the same biclusters are more functionally similar than mRNAs of different biclusters.

La Rosa et al. present a paper entitled "Alignment-free analysis of barcode sequences by means of compression-based methods" [4]. This study describes an alignment-free approach for taxonomic analyses of barcode sequences. The method relies upon two compression-based versions of non-computable Universal Similarity Metric (USM) class of distances. The authors report that this approach reproduces topological representations comparable to those of classic evolutionary methods for 94% of real barcode sequence datasets and for 100% of simulated datasets.

The paper "A subgraph isomorphism algorithm and its application to biochemical data" by Bonnici et al. [5] proposes a novel and simple heuristic subgraph matching algorithm, called RI, whose search strategy depends only on the pattern graph. By comparing its performances with three other algorithms on four graph datasets with various characteristics, authors show that it performs better than the other algorithms in terms of matching time, memory and total execution time.

### New software tools

RNA editing is an important post-transcriptional modification which alters the RNA sequence in specific sites. Many forms of editing are known affecting single residue changes or even stretches of residues. VIRGO, by Distefano et al. [6], is a web-based tool which allows a systematic identification of putative A to I editing sites in genomic sequences, based on the integration of information from UCSC, EST of NCBI, SNPs, DARNED and Next Generation Sequencing data.

D'Antonio et al. present a paper called "WEP: a high-performance analysis pipeline for whole-exome data" [7]. The authors describe a web-based tool, named WEP, for processing raw whole exome sequencing data obtained from the Illumina platform. Their contribution is a pipeline that automatizes the analysis of whole exome sequencing data, starting from quality controls of submitted short reads (produced in single or paired end) to SNP identification and variant annotation. The aim of this tool is to overcome the user's difficulty to process their own data, especially in the case of researchers of small labs with limited computational resources and experiences.

Vicedomini et al. present a paper entitled "GAM-NGS: Genomic Assemblies Merger for Next Generation Sequencing" [8], where they propose an algorithm for assembling NGS data. In particular, they developed GAM-NGS (Genomic Assemblies Merger for NGS), whose primary goal is to merge two or more assemblies in order to enhance contiguity and correctness of both. The proposed approach is capable to overcome the common shortcomings present in other assemblers. In particular, GAM-NGS is able to merge two or more assemblies in order to improve contiguity and correctness, and can be used on all NGS-based assembly projects. However, it shows its full potential with multi-library Illumina-based projects.

The article "NGS Trex - Next Generation Sequencing Transcriptome profile explorer" by Boria et al. [9] describes NGS Trex, a new pipeline system that analyses RNA-seq data: the system automates the process of investigating raw data by mapping reads to reference genomes, and also analyzes differential expression of genes and splice variants.

### Protein structure and function

The paper "Identification and analysis of conserved pockets on protein surfaces", by Cammisa et al. [10], reports a work focussed on a major and attracting subject of the structural bioinformatics, namely the prediction of ligand binding pockets on the protein surface. The authors describe an approach able to identify the most likely binding pockets among those detected by other programs for protein surface analysis (CastP in the reported case), relying on sequence conservation. The developed DrosteP tool is an optimized procedure to measure the evolutionary conservation of the residues lining the pockets. DrosteP was benchmarked on all human proteins with known 3D structure and active site annotated in UniProt [11]. On this dataset it was shown to be 81% accurate in the identification of the most conserved pocket.

Di Domenico et al. presented a paper titled "Analysis and consensus of currently available intrinsic protein disorder annotation sources in the MobiDB database" [12]. Their contribution present a thorough description of MobiDB, a recently published database of experimental and predicted disorder in proteins. In addition to descriptive statistics on the various available annotation sources, the paper reports a novel disorder consensus annotation calculation and its related weighting scheme.

### New scientific insights

Basu et al. present a paper entitled "Examples of sequence conservation analyses capture a subset of mouse long non-coding RNAs sharing homology with fish conserved genomic elements" [13]. The authors propose a pipeline to select candidate long non coding RNAs (lncRNAs) conserved among vertebrates and apply this pipeline to perform a sequence conservation analysis between mouse lncRNAs and zebrafish conserved genomic elements. Thus, they show that conservation at the sequence level can identify a subset of putative lncRNA orthologs. The similar protein-coding neighborhood and transcriptional information also provide support to the hypothesis that these lncRNAs share functional homology.

The paper by Carrara et al [14], entitled "State of the art fusion-finder algorithms are suitable to detect Transcription-Induced Chimeras in normal tissues?", evaluates the efficacy of "state of the art" fusion finders in detecting chimeras in RNA-seq data from normal tissues. The results of this study highlight a dependency of the available tools on read length, quality score and on the number of reads supporting each chimera. Thus, the paper underlines the need to carefully select the used software on the basis of RNA-seq data features.

In the paper "Logic Learning Machine creates explicit and stable rules stratifying neuroblastoma patients" [15], Cangelosi et al. develop a novel prognostic classifier of neuroblastoma patients' outcome blending existing knowledge on clinical and molecular risk factors with the prognostic NB-hypo signature. In particular, the proposed model is capable to give explicit rules that could be easily translated into the clinical setting. The results show that the proposed workflow generates a stable classifier, very accurate in predicting good and poor outcome patients.

The "Systems Biology" topic is represented by the work of Calviello et al. [16]. In this paper, the authors apply a systems biology approach to the study of the anomalous entrapment phenomenon that is supposed to take place when solutes are entrapped in very small volume compartments. The final output of this theoretical model is a table of the expected fluorescence kinetics values for different inner concentration of solutes. These data are useful to experimental researchers for planning experiments to detect anomalies in the entrapment process.

### Next meeting

The next Annual meeting of the Italian Society of Bioinformatics will be held in Udine, May 21-23, 2013. Further information about BITS 2013 will be available on our web site [1], as well as on a purpose web site to be announced.

## Competing interests

The authors declare that there are no competing interests.

## Declarations

This supplement has not been supported by sponsorship.

This article has been published as part of *BMC Bioinformatics *Volume 14 Supplement 7, 2013: Italian Bioinformatics Society (BITS): Annual Meeting 2012. The full contents of the supplement are available online at http://www.biomedcentral.com/bmcbioinformatics/supplements/14/S7
